# In vivo fertilization improved the cryotolerance and developmental ability of vitrified-warmed rat fertilized oocytes

**DOI:** 10.1038/s41598-024-76073-x

**Published:** 2024-10-15

**Authors:** Yuta Ishizuka, Satohiro Nakao, Tsutomu Kamisako, Katsuma Yamaga, Naomi Nakagata, Hiroyoshi Ishizaki, Toru Takeo

**Affiliations:** 1 Kobe Research Laboratories, Eisai Co., Ltd. 6-8- 2 Minatozima Minamimachi, Chuo-ku, Kobe, 650-0047 Japan; 2 Division of Reproductive Engineering, Center for Animal Resources and Development, Kumamoto University, 2-2-1 Honjo, Chuo-ku, Kumamoto, 860- 0811 Japan; 3 Division of Reproductive Biotechnology and Innovation, Center for Animal Resources and Development, Institute of Resource Development and Analysis, Kumamoto University, 2-2-1 Honjo, Chuo-ku, Kumamoto, 860-0811 Japan

**Keywords:** Rat, In vivo-fertilized oocyte, Cryopreservation, Genetic engineering, Genetic engineering, Embryology

## Abstract

The cryopreservation of rat embryos is useful for efficiently archiving rat resources in bioresource repositories. The cryopreserved fertilized oocytes can be quickly reanimated to rats with homozygous mutations using embryo transfer. In addition, cryopreserved rat fertilized oocytes are easier to transport than live animals. Before cryopreservation, fertilized oocytes are obtained by mating or in vitro fertilization. However, it is not clear which fertilized oocytes are most suited to cryopreservation. In this study, we performed a systematic comparison of the fertilizing ability, cryotolerance, and developmental ability of cryopreserved fertilized oocytes at the pronuclear stage produced either by mating (in vivo) or in vitro fertilization (in vitro) in SD and F344 rats. In vivo-fertilized oocytes had higher cryotolerance and developmental ability than in vitro-fertilized oocytes in SD and F344 rats. Furthermore, the fertilization ability, cryotolerance, and developmental ability of vitrified-warmed fertilized oocytes differed between SD and F344 rats. In conclusion, our study suggests that in vivo-fertilized rat oocytes were more suitable for cryopreservation. Our protocol provides an optimized system for the management of rat colonies using fertilized oocytes cryopreservation and contributes to the 3Rs principle by reducing the number of animals used for research.

## Introduction

In drug discovery research, experimental animals are essential to the evaluate medical benefits, safety, and pharmacokinetics of new drugs. In particular, rats are used for experiments such as safety evaluation because of their size, ease with which surgery can be performed, and ability to under repeated blood sampling. Rats are a commonly used animal to support the understanding of biological phenomena and human diseases. Moreover, genome editing technology has increased the values of rats as models for human disease to facilitate the development of cutting-edge therapies. Accordingly, genetically engineered rats have gone into production worldwide.

Many rat strains have been collected, preserved, and supplied to rat banks (RRRC; https:/www.rrrc.us/, Kyoto University; https://www.anim.med.kyoto-u.ac.jp/nbr/Default.aspx). In rat banks, reproductive technology is applied to efficiently collect, archive, and transport the rat resources. Recently, a procedure for in vitro fertilization (IVF) was improved to achieve these objectives^1,2^. Moreover, Nakagata developed optimized protocols for the cryopreservation of rat sperm and IVF using the sperm^3,4^. In addition, Yamaga developed the cold transport of rat sperm and IVF using the sperm^5,6^. The transport of cryopreserved or cold-stored rat sperm allows inexpensive and easy shipment among animal facilities. However, the transported sperm can only be developed into pups with heterozygotes or wild-type alleles using IVF and embryo transfer (ET). To obtain rats with homozygous mutations, the offspring is needed for mating or for IVF and ET to be performed.

The cryopreservation of fertilized oocytes and embryos is one solution to transport the rats with homozygous mutations. In rats, fertilized oocytes or embryos were produced by natural mating using female rats subjected to superovulation treatment before cryopreservation^7–12^. IVF is one of the main methods for producing fertilized oocytes or embryos based on recent improvements in rat reproductive technology. However, the cryotolerance of in vivo and in vitro-fertilized rat oocytes and the efficacy of animal production using cryopreserved fertilized oocytes derived in vivo or in vitro are not clear.

In this study, we investigated the cryotolerance and developmental ability of in vivo or in vitro-fertilized rat oocytes after vitrification and warming. In rats, the pronuclear stage embryos are available for producing genetically engineered animals by genome editing techniques and animal production and colony expansion by embryo transfer. So, the cryopreservation of pronuclear stage embryos is important in rats. First, the fertilization rate of SD or F344 rats was examined after mating or in vitro fertilization. Second, the survival rates of in vivo and in vitro-fertilized oocytes were evaluated. Finally, ability of vitrified-warmed fertilized oocytes derived from in vivo and in vitro fertilization to develop into blastocysts or fetuses was examined by in vitro culture or embryo transfer. SD and F344 rats were adapted for animal models in this study. The Crl: CD(SD) rat is a closed colony that is produced by International Genetic Standard System (Charles River Laboratory; https://www.criver.com/products-services/find-model/cd-sd-igs-sopf-rat? region=28). The F344 strain is an inbred rat for which the whole genome sequence has been determined (Kyoto University; https://www.anim.med.kyoto-u.ac.jp/nbr/seqbrowser.aspx).

## Results

*Strain-dependent differences of the fertilization efficiency in SD and F344 rats in vivo and in vitro*.

In the in vivo fertilization experiment, the copulation rates were different in SD and F344 rats (Table 1). The copulation rate of F344 rats was lower than that of SD rats.

**Table 1:** The copulation rate of SD and F344 female rats.

Strain	No. of females	No. of female rats copulating (%)
SD	24	17 (70.8)
F344	24	6 (25.0)

**Fig. 1:**
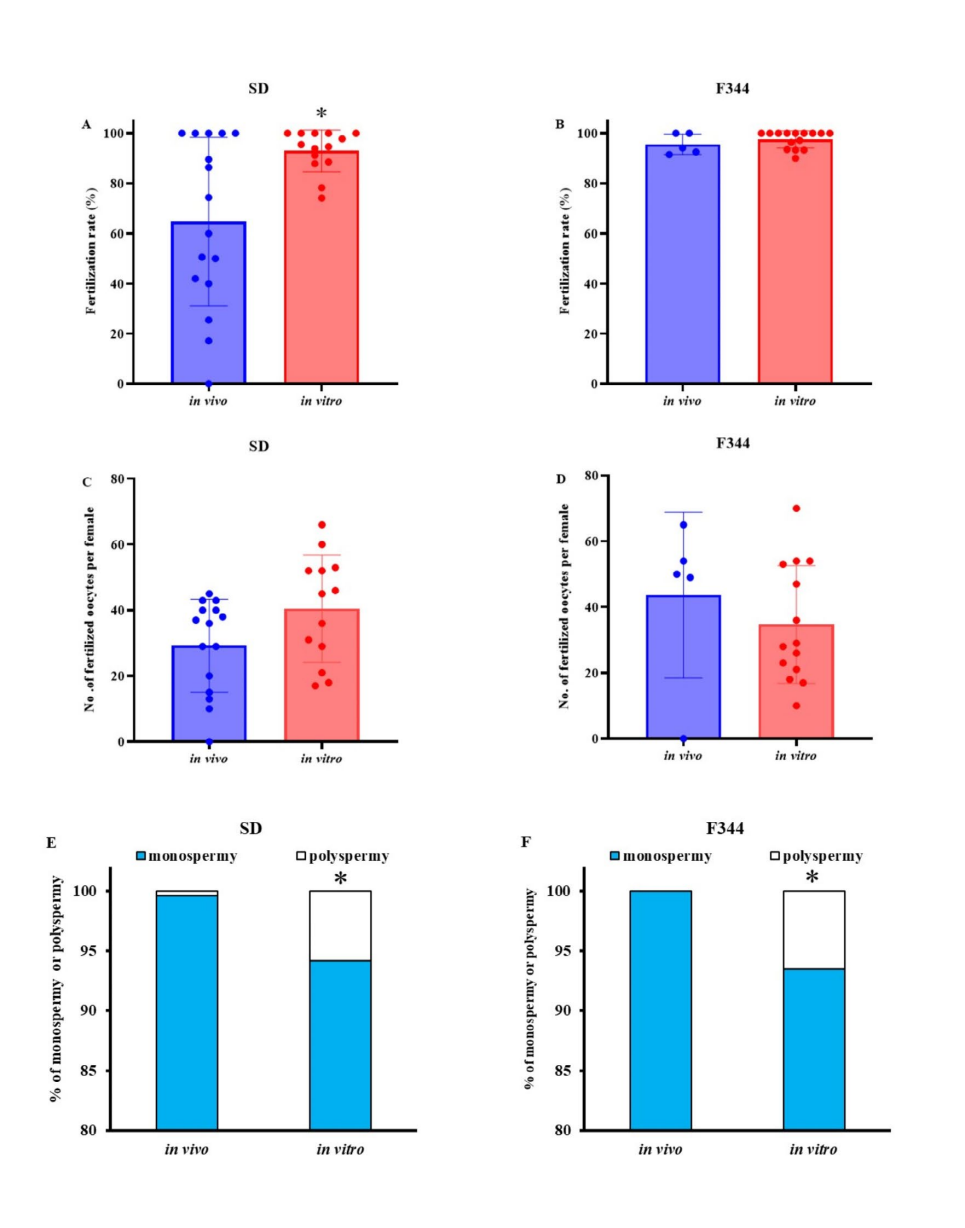
Total fertilization rate and the rate of monospermic and polyspermic fertilization in SD and F344 rats. These figures show the fertilization rate, the efficacy of fertilized oocytes collection, and the rates of monospermic and polyspermic fertilization after in vivo and in vitro fertilization in SD (**A, C, E**) and F344 (**B, D, F**) rats. **A, B**) in vivo refers to fertility by mating and in vitro refers to fertility by in vitro fertilization. The numbers of fertilized oocytes and the total numbers of fertilized oocytes and unfertilized oocytes were as follows: SD: in vivo, 465/776; in vitro, 615/663; F344: in vivo, 237/245; in vitro, 627/648). **C, D**) Efficacy of collection of fertilized oocytes in in vivo (No. of fertilized oocytes obtained from each female rat with a plug: SD rats, 17/24; F344, 6/24) or in vitro (No. of fertilized oocytes obtained from each ovulating female rat: SD, 13/15; F344, 14/15). **E, F**). The monospermic (blue bar) and polyspermic (white bar) fertilization were evaluated from observations of the pronuclei and sperm tail (more than three pronuclei and more two sperm tails were defined as polyspermy). The polyspermic rates were 0.4 ± 0.004% in SD and 0% in F344. *Values are significantly different in the comparison between in vivo and in vitro fertilization (*P* < 0.05).

In SD rats, the rate of in vivo fertilization was lower than the rate of in vitro fertilization (Fig. 1A). In contrast, rates of in vivo and in vitro fertilization were equal in F344 rats (Fig. 1B). However, there was no significant difference in the average number of fertilized oocytes obtained from each female rat by copulation (in vivo fertilization) or ovulation (in vitro fertilization) (Fig. 1C and D). In vivo fertilization led to lower rates of polyspermic fertilization than in vitro fertilization in SD and F344 rats (Fig. 1E and F). The fertilized oocytes were used to evaluate cryotolerance after vitrification and warming and the developmental ability of the vitrified-warmed oocytes was assessed after embryo culture and transfer.

### Cryotolerance of in vivo-fertilized oocytes was higher than that of in vitro-fertilized oocytes

**Fig. 2:**
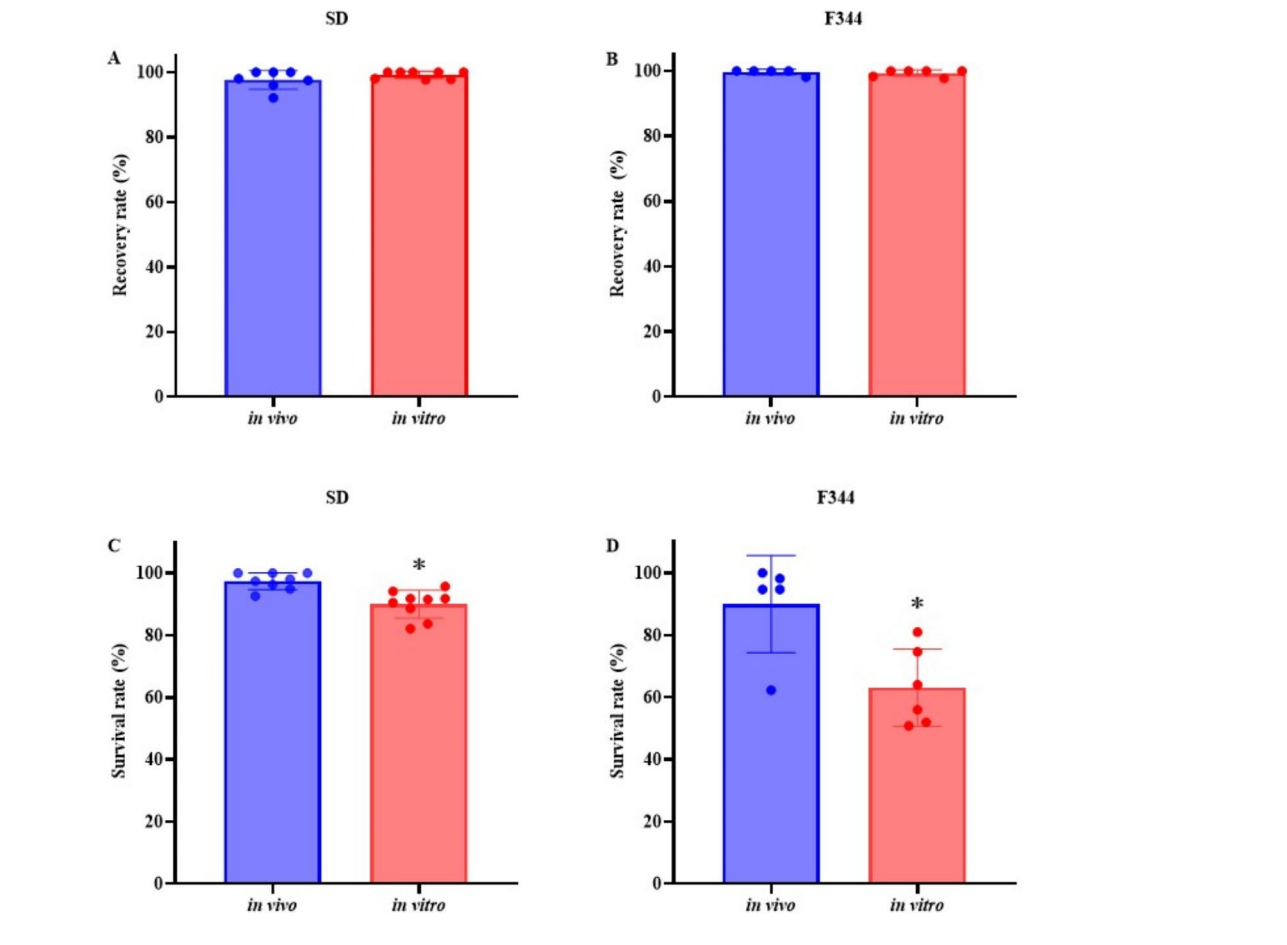
Recovery rate and survival rate of vitrified-warmed fertilized oocytes derived from in vivo and in vitro fertilization in SD and F344 rats. The recovery rate for SD (**A**) and F344 (**B**) rats was calculated as the number of collected fertilized oocytes divided by the number of vitrified embryos times 100. The survival rate of SD (**C**) and F344 (**D**) fertilized oocytes was calculated as the number of morphologically normal fertilized oocytes divided by the number of collected fertilized oocytes times 100. *Values are significantly different in the comparison between in vivo and in vitro (*P* < 0.05).

The recovery and survival rates after vitrification and warming were shown in Fig. 2A, B, C and D. The survival rate of in vivo-fertilized oocytes was slightly higher than that of in vitro-fertilized oocytes in SD rats (Fig. 2C). Similarly, in vitro-fertilized oocytes of F344 rats had a lower survival rate than in vivo-fertilized oocytes after vitrification and warming (Fig. 2D). The cryotolerance of vitrified-warmed oocytes was different between in vitro and in vivo-fertilized oocytes. After the survival rate was determined, the surviving oocytes were used for evaluation of the developmental abilities after embryo culture and transfer.

## In vivo-fertilized oocytes had higher developmental abilities than in vitro-fertilized oocytes after vitrification and warming

**Fig. 3:**
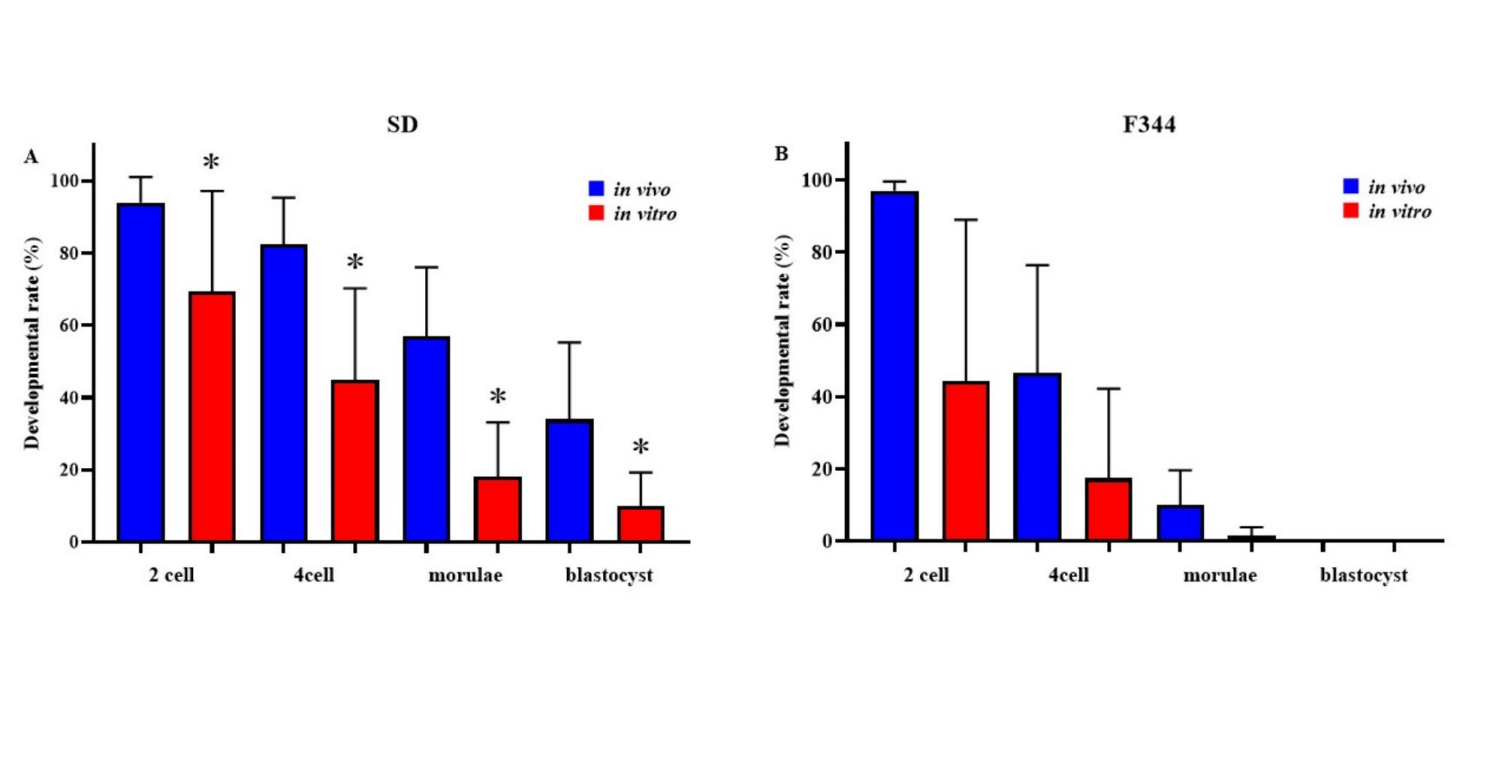
Ability of vitrified-warmed fertilized oocytes derived from in vivo and in vitro fertilization to reach each development stage. To examine the developmental ability of fertilized oocytes in SD (**A**) and F344 (**B**) strains, PN oocytes derived from in vivo and in vitro fertilization were cultured in mR1ECM. The developmental rates of each stage were calculated as the number of embryos in each stage divided by the number of cultured pronuclear stage oocytes × 100. Fertilized oocytes in the F344 strain derived from in vivo and in vitro did not develop into blastocysts. *Values are significantly different in the comparison between in vivo and in vitro (*P* < 0.05).

In embryo culture, the fertilized oocytes at the pronuclear stage after vitrification and warming were cultured to blastocysts in mR1ECM and the developmental rate was compared for in vivo and in vitro-fertilized oocytes. In SD rats, the developmental rates of in vivo-fertilized oocytes were higher than those of in vitro-fertilized oocytes (Fig. 3A). However, the in vivo and in vitro-fertilized oocytes of F344 did not develop to blastocysts (Fig. 3B).

**Fig. 4:**
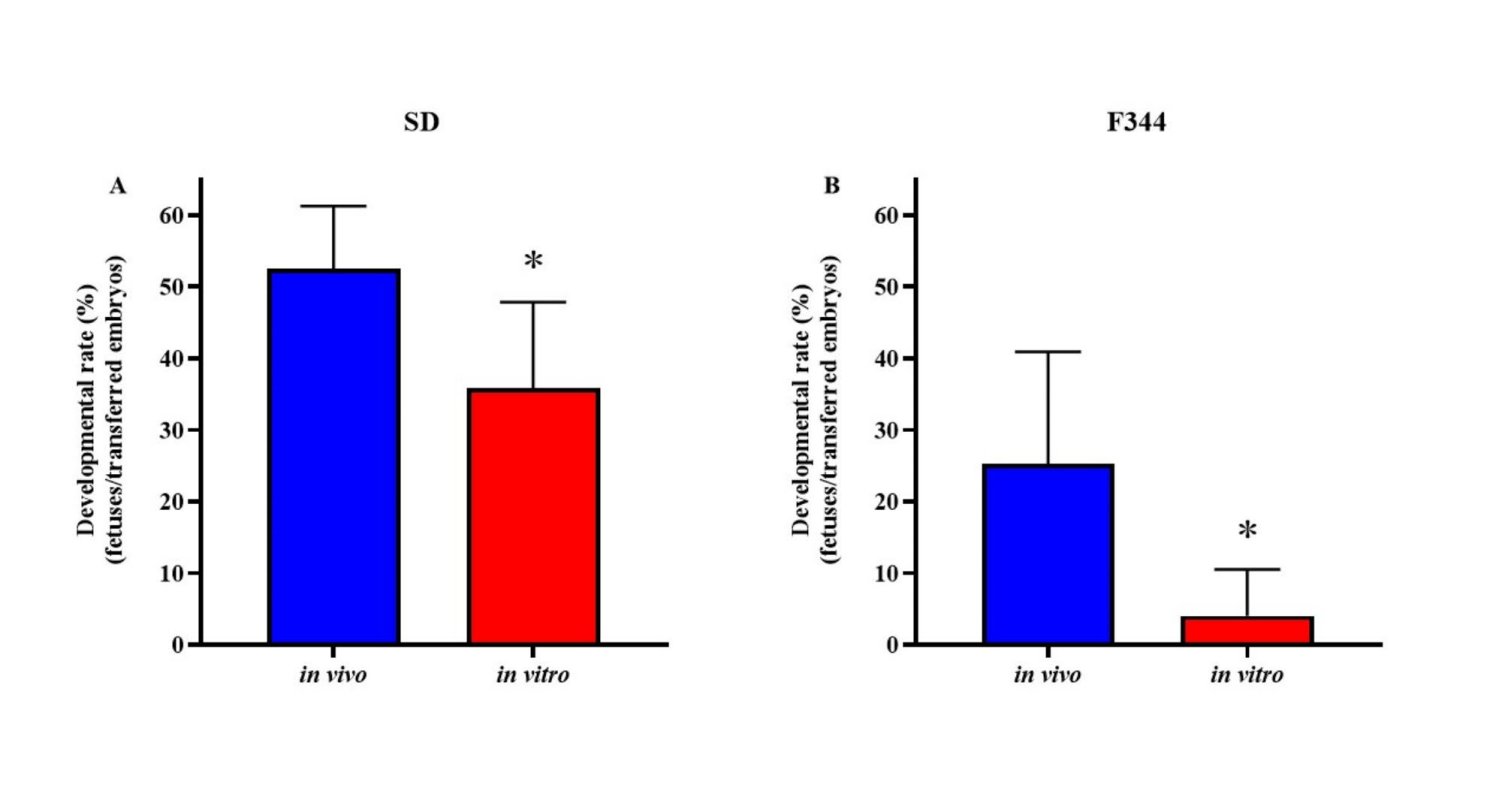
Ability of vitrified-warmed fertilized oocytes derived from in vivo and in vitro fertilization to develop into a fetus. The ability of vitrified-warmed fertilized oocytes of SD (**A**) and F344 (**B**) to develop into fetuses following embryo transfer was examined. Blue bars show the development rate of in vitro-fertilized oocytes and red bars show that of in vivo-fertilized oocytes. The developmental rate was calculated as the number of fetuses divided by the number of transferred PN oocytes × 100. *Values are significantly different in the comparison of in vivo and in vitro (*P* < 0.05).

After transferring the fertilized oocytes at the pronuclear stage with after vitrification and warming treatment to the recipient rats, normal development to the fetal stage was observed (Fig. 4). The developmental rates of in vivo-fertilized oocytes were higher than those of in vitro-fertilized oocytes in SD (Fig. 4A) and F344 (Fig. 4B) rats.

## Discussion

**Fig. 5:**
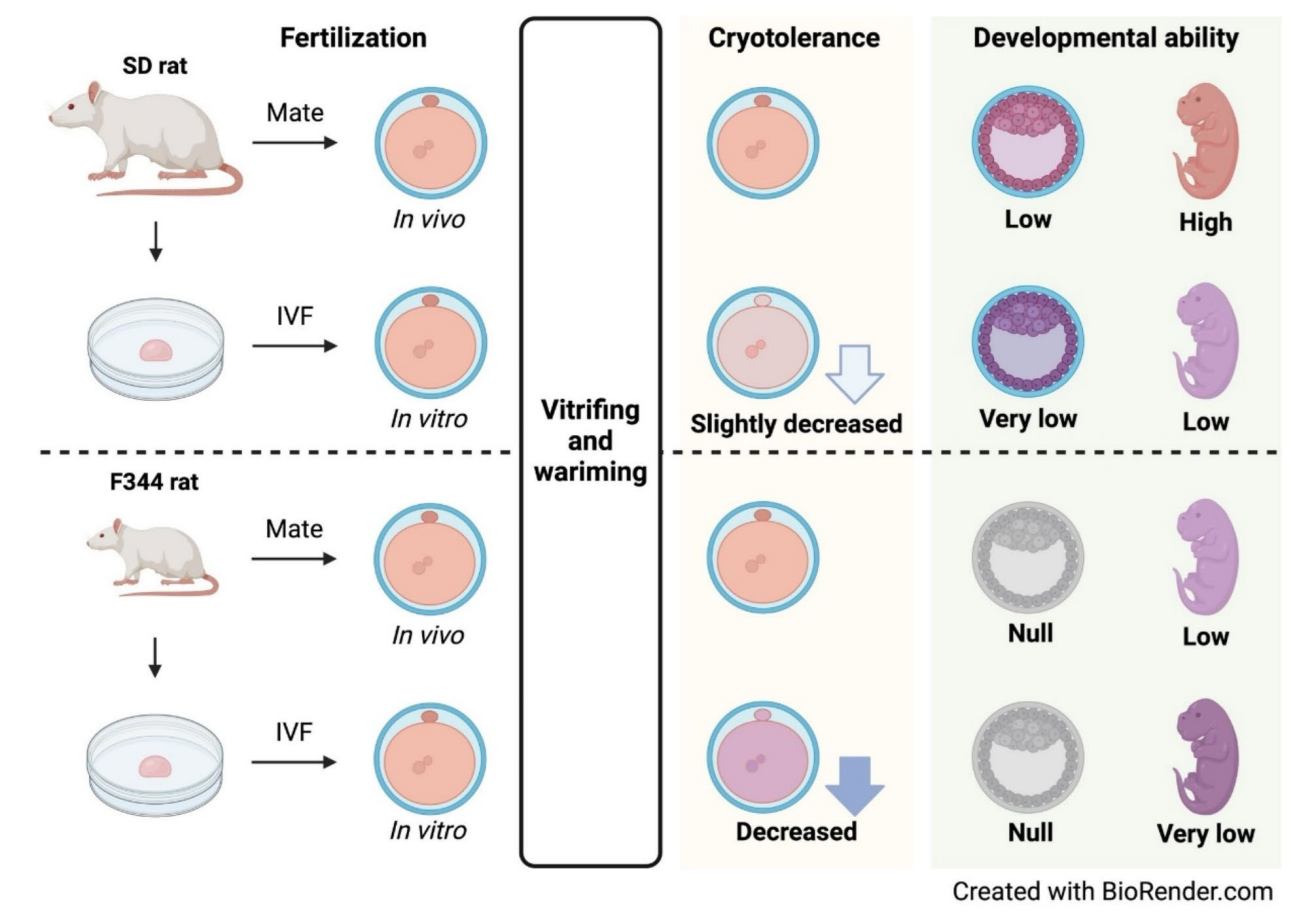
Summary of these research findings. The cryotolerance and developmental ability of in vivo and in vitro-fertilized SD and F344 rat oocytes were evaluated after vitrifying and warming. In vivo-fertilized oocytes maintained high developmental ability after vitrification in both strains. In vitro-fertilized oocytes in both strains had lower developmental abilities than in vivo-fertilized oocytes; notably, the F344 strain experienced severe freezing failure. This figure was created by Biorender (https://www.biorender.com).

Our study performed a systematic comparison of the yield of fertilized oocytes, the cryotolerance of the vitrified-warmed oocytes and the fetus productivity was compared in vivo and in vitro-fertilized oocytes in SD and F344 rats (Fig. 5). The in vivo-fertilized oocytes had higher cryotolerance and greater developmental ability than in vitro-fertilized oocytes in SD and F344 rats. In F344, in vivo fertilization is limited by the low copulation rate (25%). The different rat strains had different rates of in vivo and in vitro fertilization (SD: in vivo 64.7% vs. in vitro 93.0%; F344: in vivo 95.7% vs. in vitro 97.6%).

The cryopreservation of rat embryos is necessary to efficiently transport and archive homozygous mutations of rat resources. We demonstrated efficient protocols for preparing in vivo-fertilized oocytes, cryopreserving fertilized oocytes, and performing embryo transfer using these oocytes in SD and F344 rats. A previous study showed that the vitrified-warmed fertilized Wistar rat oocytes produced by in vitro fertilization had a low survival rate (41.2%) and a low birth rate (10%)^13^. Our protocol increased the low survival and birth rates of vitrified-warmed fertilized oocytes using in vivo-derived fertilized oocytes from SD and F344 rats. The protocol provides a practical solution to manage rat strains without breeding the colony and contributes to a reduction in the number of animals used for research.

The developmental ability to fetuses of in vivo-fertilized vitrified/warmed oocytes was higher than that of in vitro-fertilized vitrified/warmed oocytes in SD and F344 rats. In this study, we produced fetuses from in vivo-fertilized oocytes after vitrification and warming. The protocol for in vivo fertilization also increased the yield of two-cell embryos, four-cell embryos, morulae, and blastocysts after embryo culture in SD rats. These embryos will be able to applied to cryopreservation with various stage embryos^10^, animal production by embryo transfer with 2-cell embryos^14^, and genetic modification by genome editing technology with 1-cell and 8-cell embryos^15,16^, blastocyst complementation technology with blastocyst embryos^17^, and the preparation of embryonic stem cells with blastocyst embryos^18^.

The cryotolerance of fertilized oocytes was different between in vivo and in vitro fertilization in SD and F344 rats. The fertilized oocytes of F344 rats were more sensitive to damage from the vitrification and warming procedures than that of SD rats. Similarly, the difference in cryotolerance between in vivo and in vitro-derived embryos is reported in some animal species such as bovine, ovine and holstein^19–22^. The damage from the vitrification and warming procedures could be caused by the toxicity of cryoprotectants, osmotic stress^11^, ice crystal formation^23,24^, and oxidative stress during recovery^25^. To improve the survival rate of in vitro-fertilized oocytes, further experiments are required to examine the effect of rapid warming^11,12^ and to identify the critical steps impacting damage during vitrification.

Some technical limitations exist regarding in vivo fertilization. The variation in fertilization rates was observed after mating in SD rats. The unstable rates of in vivo fertilization may be caused by variations in the number of sperm reaching oocytes in ampulla^26–28^. In our previous study, synchronization of the timing of ovulation and copulation enhanced the in vivo fertilization rates in mice^29^. Therefore, adjusting the timing of the mating step may stabilize and enhance the in vivo fertilization rates in SD rats. However, the low success rate of copulation (25%) occurred in F344 rats. In this study, we demonstrated that there was a difference in copulation rates between SD and F344 rats. According to the previous report, there was a difference in the copulation rates among rat strains of SD, Long-Evans, and Wistar^30^. On the other hand, Mochida et al. have reported a high copulation rate in F344 rats^31^. Their study used adult female rats and administered only anti-inhibin monoclonal antibodies. The condition of superovulation treatment may affect the copulation rate in F344. In addition, oxytocin regulated the sexual behavior in male and female rats^32^. The administration of oxytocin may be effective in improving the copulation rate.

**Fig. 6:**
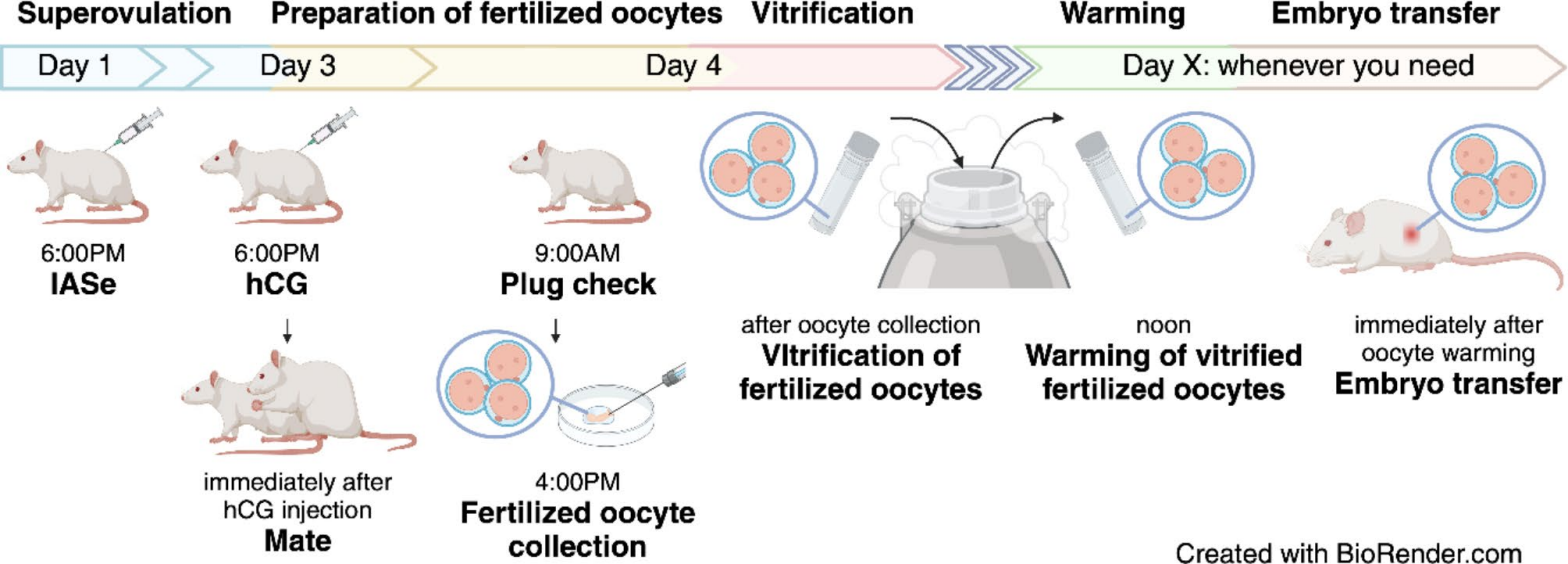
Protocols for producing rats with cryopreserved oocytes fertilized in vivo. In this study, we systematically compared the yield of fertilized oocytes, the cryotolerance of the vitrified-warmed oocytes, and the fetus productivity between in vivo and in vitro-fertilized oocytes in SD or F344 rats and demonstrated efficient protocols for preparing in vivo-fertilized oocytes, cryopreservation, and embryo transfer. This figure was created by Biorender (https://www.biorender.com).

In conclusion, we demonstrated that in vivo-fertilized rat oocytes had a higher cryotolerance and developmental ability. Our protocol (Fig. 6) provides an optimized system for the management of rat strains based on the 3Rs principle by reducing the number of rats used for breeding.

## Methods

### Animals

Crl: CD(SD) (SD: Sprague Dawley) and F344/DuCrlCrlj (F344) rats were obtained from Jackson Laboratory Japan, Inc (Kanagawa, Japana). Male rats, at 11–18 weeks of age, were mated with female rats at 4–5 weeks of age. Pseudopregnant Slc: SD rats were purchased from Slc Japan (Shizuoka, Japan) and were used as recipients for embryo transfer. All animals were bred at animal facility authorized by the Japan Health Sciences Foundation. The rats were kept under constant temperature (23℃ ± 3℃), humidity (55% ± 5%) and a 12-hour light/dark cycle (lights switched on at 8:00 and off at 20:00). All animal studies were approved from the Animal Ethics Committee at KAN Research Institute (currently Kobe Research Laboratories, Eisai Co., Ltd.) (22-C-0141-001, 22-C-0141-002) and conducted in accordance with their Laboratory Animal Welfare guidelines. This study is reported following ARRIVE guidelines.

### Media and reagents

Calcium enhanced human tubal fluid (mHTF) was used for the sperm preincubation medium and the fertilization medium^33^. Reagents of 1 M dimethyl sulfoxide (DMSO), 2 M DMSO, 1 M acetamide, 3 M propanediol (DAP213), and 0.3 M sucrose in phosphate-buffered saline 1 (SPB1) were used for vitrifying and warming fertilized oocytes^34^. SPB1 was purchased from ARK Resource Co., Ltd. (Kumamoto, Japan). Potassium Simplex Optimized Medium (KSOM) and modified rat one-cell embryo culture medium (mR1ECM) were used for embryo culture in vitro^35,36^. The medium for manipulating fertilized oocytes was phosphate-buffered saline 1 (PB1) medium^37^. Superovulation was induced by PMSG (pregnant mare’s serum gonadotropin (serotropin; Aska Animal Health Co., Ltd. (Tokyo, Japan)), IAS (inhibin antiserum), and hCG (human chorionic gonadotropin (gonatropin; Aska Pharmaceutical Co., Ltd. (Tokyo, Japan)).

## In vivo fertilization by mating

To induce superovulation, immature SD and F344 female rats were injected with 30 IU PMSG and 0.2 mL IAS in SD rats or 15 IU PMSG and 0.2 mL IAS for F344 rats, and were injected 30 IU hCG in SD rats or 7.5 IU hCG in F344 rats at 48–50 h after the injection of PMSG and IAS^4,38^.

Male rats were bred individually in cages before mating. After hCG injection, female rats were co-housed and mated with male rats of same strain. The vaginal plug was checked the morning after mating and the copulation rate was recorded. At 22 h after the hCG injection, the oocytes were collected from the euthanized female rats after copulation by flushing the oviduct. The collected oocytes were washed in a 50 mL volume of PB1 medium three times. Then, the fertilization rate was calculated as the number of fertilized oocytes at the pronuclear stage divided by the total number of fertilized and unfertilized oocytes multiplied by 100. The determination of fertilized oocytes was performed in accordance with a previous report^39^.

## In vitro fertilization

In vitro fertilization was performed using fresh sperm in accordance with the modified procedure of Nakagata et al.^3^. To collect fresh sperm, mature rats were euthanized by the inhalation of carbon dioxide. The cauda epididymides were collected from the euthanized rats. Sperm were collected from the cauda epididymides and transferred into a 400 µL drop of mHTF. In addition, the concentration of sperm was calculated using a hemocytometer (Erma; Tokyo, Japan). The sperm suspension was transferred into a 200 µL drop of mHTF (fertilization drop) and the final concentration was adjusted to 400–500 sperm/µL. Subsequently, sperm were incubated at 37℃ in 5% CO_2_ for 2 h.

Superovulation was performed following the same procedures by in vivo fertilization. At 14–16 h after the injection of hCG, female rats were euthanized by cervical dislocation and the oviducts were collected. The oocytes were collected from the oviducts and were transferred into the drop of mHTF including sperm (the fertilization drop). The sperm and oocytes were incubated at 37℃ in 5% CO_2_ for 6 h.

Oocytes at the pronuclear stage were then washed in a 50 mL drop of mHTF. The fertilization rate was calculated as the number of fertilized oocytes at the pronuclear stage divided by the total number of fertilized oocytes and unfertilized oocytes multiplied by 100. Fertilized oocytes were determined in accordance with a previous report^39^.

### Vitrification and warming of fertilized oocytes

The fertilized oocytes obtained from in vivo or in vitro fertilization were cryopreserved by vitrification, with some modifications. The fertilized oocytes were transferred to a drop of approximately 100 mL drop of 1 M DMSO solution and were equilibrated at room temperature for 5 min. Thereafter, 5 µL of 1 M DMSO solution including the fertilized oocytes was transferred into a cryotube, and the cryotube was placed on ice for 2 min. After equilibration for 2 min, a 50 µL aliquot of DAP213 solution was added into the cryotube. Then, the cryotube was immersed and preserved in liquid nitrogen until warming (at least 5 days).

To warm the vitrified oocytes, the cryotube was removed from liquid nitrogen and placed at room temperature (23℃) for 30 s. Then, a 900 µL aliquot of SPB1, warmed to 37℃, was added to the cryotube and mixed gently by pipetting. The fertilized oocytes were collected and were washed in a 50 mL drop of PB1 medium. At this time, the morphological normality of the fertilized oocytes was observed and the survival rate was calculated. The morphological normal oocytes were used for the subsequent in vitro culture and embryo transfer.

### In vitro culture and embryo transfer

The portion of vitrified-warmed fertilized oocytes were transferred into a 50 mL drop of KSOM medium. The fertilized oocytes were incubated at 37℃ until they developed to a two-cell embryo. The two-cell embryos were transferred to a 50 mL drop of mR1ECM and were incubated at 37℃ for 96 h. At 48, 72, and 96 h after incubation by mR1ECM, the embryos were observed at the four-cell, morula, and blastocyst stages. The developmental rates at each stage were calculated as the number of embryos at each stage divided by the total number of the vitrified-warmed fertilized oocytes multiplied by 100.

The another vitrified-warmed fertilized oocytes were used for embryo transfer. The fertilized oocytes were transferred into both oviducts of pseudopregnant rats (approximately 20 oocytes/oviduct) on the day a vaginal plug was found. After 21 days, the number of fetuses were observed via cesarean section. The rate of fetal development was calculated as the number of fetuses divided by the total number of the transferred fertilized oocytes multiplied by 100.

### Statistical analysis

Statistical analysis was performed using an unpaired Mann-Whitney (nonparametric) test by GraphPad Prism (version 9.3.1). The results in all experiments were shown as mean ± standard deviation. P-values less than 0.05 were considered statistically significant.

## Data Availability

The authors declare that the data supporting the findings of this study are available within the paper and its supplementary files.
